# Science and a Future Life

**Published:** 1890-04-12

**Authors:** 


### The Hospital

A Weekly Journal of

**Science, Medicine, Nursing, and Philanthropy.**

*For Hospitals, Asylums, Medical Practitioners, Students, Nurses, Families, Parishes, Congregations, and the Charitable Public.*

Vol. VIII.—No. 185.] [April 12, 1890.

### SCIENCE AND A FUTURE LIFE.

The Royal Society of Great Britain gives promise of recovering its character for breadth of view, and its capacity for both speculative and practical reasoning. Two very energetic and eminent personalities among its fellows, whom we need not name, have for a good many years not only succeeded in narrowing the range of their own thought, but also in bringing almost into ridicule the wider regions of philosophic inquiry which have proved so irresistibly attractive to the most powerful and adventurous minds in every age since the very birth of philosophy. But these two, we are glad to think, have had their day. Great as have been the services they have rendered to biology and chemistry, those services have been largely discounted by the curious incapacity displayed by both of them, for the noble idealism which owns Plato for its parent, and for the nobler practical ethics which originated with, and derive authority from, the teaching and life of Christ. It is a calamity for the world when men who gain the public ear, and influence thought and practice, are dwarfed and stunted in the nobler parts that characterise the perfect and typical man. A creature that is at one and the same time both an expounder of principles and an object lesson, has in him a potentiality which is practically immeasurable; and hence it is that those who ardently desire the best interests of the race always regard with anxiety the appearance of new stars in the intellectual firmament, and with dread the familiar luminaries that shine with a light some of whose rays are of doubtful value.

Sir George Stokes, the present President of the Royal Society, is a man of another mind. At the Finsbury Polytechnic, a few days ago, he spent an hour in considering with the students the question of a future life. Now the question of a future life is at least as important as that of to-morrow's dinner; and yet, whilst everyone except the most shiftless will be miserable if to-morrow's, and a good many to-morrows', dinners are not provided, it is only here and there an individual who makes serious provision for that voyage which is to transfer him from a world he knows to a world where all is strange. Sir George Stokes made it very apparent that his views on the subject were neither merely academic nor used for a merely academic purpose. If we read him aright, he wished to look at the question scientifically; but the science had for its object a practical result. We have often in this journal expressed the conviction that science which has no relation to practice is worthy of much less regard than that which is pursued with practical objects. There is the same relation between the scientist who affects to despise practice and the scientist who is enthusiastic for practical ends, as there is between the child who makes mud pies and the invaluable cook who provides an appetising and invigorating dinner; and the mind of the scientist "for the sake of science" is often as vain and inconsequent by comparison with the practical man's, as is the mind of the maker of mud pies compared with that of the altogether admirable cook. Sir George Stokes, it is evident, is of opinion that the future life is eminently a practical as well as a speculative question.

A good many members of the general public seem to be of opinion that Sir George has said something damaging to religion in expressing the conviction that there is not much, if any, evidence in favour of the theory that every person born is necessarily immortal; that is, that the human soul is immortal by virtue of its own nature. Whether it is or is not there are no means of showing. Sir George Stokes does not discuss the problem in this abstract way. He does not know, any more than Socrates or Marcus Aurelius knew, whether or not the soul is immortal; but he does know that a great religion, the greatest the world has ever seen, teaches the doctrine, not of the necessary immortality of the soul, but of a future life. He knows, moreover, that that religion does not rest its teaching on a speculative or academic basis, but on the basis of definite facts. It offers that kind of evidence for its position which the law courts demand, not that kind which is current in debating societies.

Sir George Stokes made his hearers understand, and the readers of newspapers as well, that the first and last line of defence, or of attack, in the Christian argument for a future life, is the actual resurrection of Jesus Christ from the dead. This, as everyone knows, is given to the world as a well-attested historical fact in certain classical writings familiarly known to us as the "New Testament." Sir George Stokes appears to find no peculiar difficulty in believing the historical statements of the books of the New Testament. It is in truth rather an unequal conflict when law court evidence and debating society evidence meet for a trial of strength. The debating society witnesses say such and such a thing "cannot in the nature of things be true, because it is not possible"; the law court witness says, such and such a thing "is, and must be true, because I saw it with my own eyes, heard it with my own ears, and handled it with my own hands." No sane man will for a moment undervalue the trained critical ability and the enormous industry of a Strauss; nor will a mind of the least openness and literary appreciation fail to admire the genius of a Renan or a Matthew Arnold. On the other hand, what the witness-box demands is not the opinions of experts but the testimony of eye-witnesses. One actual eye-witness, especially if he be a person of undoubted probity, is worth ten thousand opinions of critics, however distinguished they may be, who only read the story of events many years after they have transpired. Out of date as Paley may be held to be by the superior persons of the present day, he presents a case for the fact of the resurrection of Christ which Strauss, at any rate, has not succeeded in shaking. Sir George Stokes appears to attach much importance to Bishop Westcott's "Gospel of the Resurrection." Of that work it is not too much to say that it is as scientific in method as the works of either Strauss or Renan, and much more scientific in detail. It founds upon testimony, and takes nothing for granted. Those who wish to see what honest and learned constructive criticism has to say on the striking and unique circumstances of the rise of Christianity will do well to possess themselves of Westcott's "Gospel of the Resurrection." No man, whether scientific or unscientific, can put time to better use than by spending his leisure for two or three months in careful study of that book.

It is no part of our business to hold a brief for the Christian faith, but it is our business to stand for the defence of science; to insist upon the value of the inductive as compared with the *a priori* method of procedure, and to maintain the incomparable superiority of the testimony of trustworthy eye witnesses to the evidence of experts who know nothing of the facts, and who evolve their dicta from their own consciousness. The progress of science is in one aspect the measure of the development of man. It is incumbent upon every man to watch that progress and the leaders of it, and if he see them go out of the way to raise a warning, and, if need be, a threatening voice, to drive them back again to the right path. Such a question as that of a future life for man is eminently one with which a noble and worthy science will concern itself. But then it must proceed by scientific methods. If no man will decide *a priori* what the character of the nervous system may be in a marine animal, the like of which he has never before seen, will any man who has even a small measure of the truly scientific temper decide *a priori* whether it is or is not a fact in the experience of the universe that persons who have died have in some instances entered upon other modes of life, and have maintained or resumed consciousness in their own proper persons? True science is the school dame who corrects all her pupils, but to that particular child of man who, standing in the middle of the floor, declares aloud what can and what can not be possible in the infinite regions that lie beyond his ken, she gives a fool's cap that will never wear out, and assigns the fool's corner for a perpetual possession. Sir George Stokes has given new courage to many men and women who devote themselves to the moral enlightenment and elevation of their fellows. Other eminent scientists would do well if, before setting forth their large generalisations, they would consider them in all their possible bearings. "Let truth be spoken though the heavens should fall;" but learned persons who have opinions that seem to be opposed to high moral sentiment and progress should lock them away in the secret cabinets of their minds until they have verified them beyond the possibility of doubt. Practical wholesomeness of life among the masses of men is of infinitely greater importance than the abstract speculations of the few.

